# Managerial competences in public organisations: the healthcare professionals’ perspective

**DOI:** 10.1186/s12913-020-05179-5

**Published:** 2020-04-15

**Authors:** Simone Fanelli, Gianluca Lanza, Cristiana Enna, Antonello Zangrandi

**Affiliations:** Department of Economics and Management, University of Parma, Via J. F. Kennedy, 6 – Parma, Italy

**Keywords:** Managerial competences, Hybrid management, Manager-physician, Human resource management, Healthcare organisation, Italy

## Abstract

**Background:**

Human resources play a critical role in encouraging efficient performance within organisations, especially for public healthcare organisations, where competences of staff are key aspects of the quality of services provided. In this context, the enhancement of competences are strategic objectives for Human Resources Management (HRM) in order to achieve excellent and lasting results. However, competences of healthcare professionals are both clinical and managerial. This study identifies specific managerial competences perceived as crucial by healthcare professionals in order to improve their performance and develop suitable HRM practices.

**Methods:**

The research methodology was divided into three main phases using mixed methods, commencing with literature review to identify the initial framework about managerial competences. Focus groups were then used to discuss evidence from the literature. Feedback from focus groups was used to draft the final questionnaire. Finally, the answers to the questionnaire were analysed through statistical software.

**Results:**

The results show that managers and professionals share a view of what specific managerial competences for healthcare organisations should be. Main competences are: quality evaluation based on outcomes; enhancement of professional competences; programming based on process management; project cost assessment; informal communication style; and participatory leadership.

**Conclusions:**

Although the issue of managerial skills in healthcare is widely discussed in literature, findings are often fragmentary. Our work includes a systematic literature review useful for more empirical studies. Furthermore, our results can support public managers who want to set up positive HRM practices for healthcare professionals.

## Background

Human Resources Management (HRM) is a system that guarantees the effective use of knowledge, competences, abilities, and other characteristics possessed by individuals aiming for the achievement of an organisation’s goals. For this reason, the topic of HRM has been very widely debated in literature since the post-industrial period, when it was highlighted that productivity is increasingly based on the knowledge, skills, and abilities of the trained human intellect [1]. These aspects are particularly evident in public organisations since the assets that incur the most costs are an organisation’s employees and because the production and quality of services depend directly on employee contribution [2]. Nevertheless, with the exception of the aspect of employee/industrial relations, the public sector compared to the private sector [3] has been neglected by HRM research. Research has however found that there are at least four main reasons for focusing on HRM in the public sector [4]. These include: 1) the lack of attention given to the public sector context in the HRM literature; 2) the importance of public sector services and the role of human resource in delivering these services; 3) the level of public investment in civil services and the need for agencies to maximise this investment; 4) the scale of workforce-related challenges confronting public sector agencies.

Historically, HRM practices in the public sector followed a traditional model of personnel administration, in which a bureaucratic employment policy accompanied Weberian practices and principles of rule-governed rational action. However, results widely perceived as unsatisfactory and the awareness of a new management approach based on the responsibility and efficiency of personnel called this system into question [5]. The introduction of New Public Management (NPM) shifted the emphasis in the public sector from administration towards management in order to achieve efficiency, effectiveness and quality of care. These new business practices include new ways of managing public sector employees, and HRM was thus included in the public sector reform agenda [6]. Today the traditional notions of career service, stable and lifelong employment and service-wide employment conditions have been challenged by principles of NPM.

An important cadre of the public sector workforce that has been especially affected by NPM are the many professions represented in public sector organisations. Evetts and Burchner-Jeziorska define a “professional” as an individual who possesses knowledge and skills designated by a professional body, usually in conjunction with universities and/or professional bodies as well as the government, and controlling entry to a profession [7]. These characteristics allow professionals greater autonomy in making decisions in the workplace and an exclusive identity that establishes boundaries between themselves and others. It is this power which has enabled them to challenge and even violate managerial directives in ways in which other employees cannot [8]. Several authors claim that the HRM practices inspired by NPM have in fact failed in public organisations where there is a large proportion of professionals [3, 5, 9]. For such organisations, it is necessary to implement innovative HRM systems that enhance the characteristics of professionals, i.e. their competences and autonomy. Nevertheless, although in literature the HRM practices that enhance competences are considered innovative [10, 11], there are few studies in this direction [2, 12]. In this scenario, the first stage for developing successful HRM is knowing which competences are perceived as crucial by professionals in order to improve performance. This aspect is preliminary for all stages of the human resource cycle: which competences should be recruited, developed, evaluated and rewarded? Our research aims to answer this question with specific reference to the public healthcare sector, where the issue of competences is a particularly critical aspect for HRM practices [2, 13]. However, the issue of skills of healthcare professionals is complicated, because they are required to have both clinical and managerial competences [14]. The need for healthcare organisations to search for increasingly efficient and efficacious management has led to a shift from a model of professional bureaucracy, characterised by professionals outside the administrative hierarchy, to a model where managerial competences are required from clinicians [15]. In this new model of organisation, clinicians are required to have transversal competences. Alongside technical professional skills, they should possess management skills for managing and enhancing resources. Thus, when healthcare organisations design their HRM practices, they now need to take into consideration the new role of the clinician in the healthcare process as well as the development of new competences required to perform the role played in the organisation. From this perspective, the present study focuses on managerial competences required by professionals to fill the role of manager-clinician.

Our study was conducted in Italy. The Italian National Health System (NHS) was established in 1978, inspired by the United Kingdom NHS. It is predominantly public and characterised by professionals who possess both clinical and managerial skills. These characteristics are common to most healthcare systems in industrialised countries, as shown in the next section of this paper, and for this reason our findings can be generalised [16]. We contribute to the literature on HRM practices in healthcare sector. The results are useful to all those organisations that want to invest in human resources to increase the quality of their performance.

### Managerial competences: geographical framework

The shift to a model relying on managerial competences of clinicians has occurred in most industrialised countries. In the United Kingdom, for example, the Thatcher reforms ushered in change within the traditional model of public services [17]. The 1983 *Griffiths Report* in fact recommended that hospital physicians should take responsibility for management together with clinical autonomy. In Denmark, the first attempt to strengthen internal hospital management in 1984 occurred with a *White Paper* on the productivity of hospitals, which contains recommendations on the “modifications to management” model [18]. In France, the idea of strengthening hospital management appeared as early as 1983, although there was no decisive move toward the method until 2002. In 2007, the *Hospital Plan* reorganised units into centres each with their own budget, utilising a model similar to the British one [18]. In Italy the 1992 reform stated that healthcare organisations were legally independent entities and increasing attention was paid to costing, management, and efficiency. The new role of Chief of Unit came into being; this individual is responsible for running and organising the structure, managing human resources, managing clinical outcomes, planning and scheduling projects, and overseeing financial, technical, and administrative targets. In the United States, the shift included various reforms, which affected both teaching and the methods of assessing student healthcare skills. The Joint Commission requires specific certification for suppliers of healthcare for the accreditation of an institution. These changes were significant in terms of professional autonomy, given that the conflicting forces of the market and legislation are capable of undermining the medical profession. Taking responsibility for management is a highly complex matter in any case, although it has long been seen simply as an administrative activity specific to the medical profession [19]. Promoting and enhancing managerial competences is therefore a significant challenge, and today these skills are often poorly developed among healthcare professionals [20]. There are also studies finding that clinicians believe they have not received adequate training for these managerial roles [21]. The blame for this lies partly with curricula of medical schools, which have largely failed to offer sufficient training.

### Managerial competences: literature framework

The terms “leadership” and “management”, are used with reference to clinical personnel to describe managers who combine a clinical professional background with managerial competences and responsibilities. As fields of study, medicine and management follow different types of logic, and a manager with a clinical professional profile needs to be able to shift from one field to another. Dual responsibility in clinical practice and management entails modifying the chain of command and generates changes with regard to both organisational configuration and professional responsibility [22]. This type of management model reached Italy from the United States during the 1990s, and aimed to assign every Chief of Unit budget, financial, and HRM responsibilities, and to promote a new type of divisional model. The model spread worldwide, involving providers in many other countries, such as Canada and the United Kingdom, where the term “manager-clinician” became widespread [18]. The term also spread to Australia, New Zealand, Scandinavian countries, Italy, France, Germany and the Netherlands [15, 23]. The position of the manager-clinician, and the interpretation of his or her role within the organisation is not free from issues. He or she, after all, does not possess the preparation or background for the role, and potential problems may reflect the absence of management training, capacity, time, and personality. These aspects generate further issues involving professional identity, lack of role awareness within the organisation, and can entail poor communication with other group members [24]. These elements partially reflect the professional development of the manager-clinician, which often lies outside a continuous program based on a model of competences.

The literature on the competences of the manager-clinician is limited and partial, and provides no mutually agreed-upon definition of the role [25]. Furthermore, current studies supply generic and partial indications regarding managerial competences without stating the specific competences necessary for a manager-clinician [26]. Nevertheless, although there is no consensus as to what is required, the concept of key competences is discussed in various studies, which find they vary according to the level of management and respective local context [26]. The Healthcare Leadership Alliance describes five competency domains common among all practicing healthcare managers [27]. Other studies identify managerial competences necessary for clinical governance in different countries. One study conducted by five Canadian medical schools identified eight roles of a clinician: medical expert, communicator, collaborator and colleague, health supporter, learner, manager, researcher, and clinician as an individual [28]. Similarly, a United States study of 100 clinicians identified aspects of their role to be: possessing the required capacity for communication, guaranteeing quality, and managing human resources. This study noted, however, that this list is not exhaustive and that other key areas of management may have been overlooked [29]. The American College of Preventive Medicine also published a definition of four main managerial competences for clinicians: supplying health care, managing costs, managing elements of the organisation, and possessing legal knowledge [30].

In conclusion, there are many studies on managerial competences in the field of health, but there is no shared vision regarding the exact competences required; most importantly, there is an absence of studies exploring the specific competences required of a manager-clinician. This study thus builds on the domains of managerial competences identified by the literature to date, and aims to identify applicable competences that are both determinant for the healthcare sector and necessary to improve organisation performance. It is essential to identify these competences in order to enact policies for their development and to make the HRM system capable of developing and enhancing them.

## Methods

The research methodology was divided into three main phases using mixed methods, commencing with literature review to identify the initial framework about managerial competences. Focus groups were then used to discuss evidence from the literature. Feedback from focus groups was used to draft the final questionnaire. Finally, the answers to the questionnaire were analysed through statistical software.

Health service research includes investigation of complex processes and systems, and may necessitate both qualitative and quantitative forms of data [31]. Furthermore, mixed method research studies draw upon the strengths of both qualitative and quantitative approaches. In our study, qualitative methodology is applied to explore the vast world of managerial competences and to identify the key concepts; and these findings are then measured in an online survey quantitatively. This methodology was used in previous studies [32], and is defined by Fetters and colleagues as “exploratory sequential design” [31].

### A systematic review of the literature

First of all, a Systematic Literature Review (SLR) was carried out to define the domains of managerial competences in the healthcare sector (topics). We followed the approach used by David and Han [33] and Newbert [34]. The databases used were *Scopus* and *Ebsco Host*. We limited the search period to 1985–2017. This period was selected because clinicians started to be increasingly involved in the management of healthcare organisations in the second half of the 1980s [35]. The keywords applied as filters were: (*“managerial competence”* OR “managerial skill*” AND health*); (“*clinical manager*” OR “*doctor manager*” OR *manager-clinician* OR *manager-physician* AND *competence* OR skill*). We deleted duplicate articles from the search, performed on two different databases, and we read abstracts of the remaining papers. In this step, two researchers independently assessed the relevance of the articles for our aim. The articles were read in their entirety, and those that proposed a systematic categorisation of competences were selected for our research.

### Focus group and questionnaire

In the second step, the research was conducted through focus group discussion (FGD). The aim of the FGD was to gather collective views on the topics of competences for healthcare management in order to identify the specific managerial competences for each topic. Ten healthcare management educators were invited to a single location to participate in the FGD. The participants were selected from a group of people who have skills and experience in the field. During the FGD, conducted in March 2018, participants were given the list of the topics emerging from the SLR. Within each topic, participants were asked to identify the specific competences required of the manager-clinician. Each participant noted the specific competences for each topic on a piece of paper, and then all competences were written up on a whiteboard to be shared and discussed. The discussions on each topic and each specific competence were moderated by one of the researchers, recorded, transcribed verbatim, and analysed using templates. Over 7 h of discussions were conducted. The main managerial competences emerging from the FGD were used to define the key items of the questionnaire (see Additional files). Moreover, in each topic, we grouped the managerial competences into two sections according to how each competence is used in practice. These sections were also discussed and shared by FGD participants.

### Survey and quantitative analysis

Finally, the questionnaire was administered online to about 1500 healthcare workers in Italy over a period of 6 months (April 2018–September 2018).

All survey recipients work in public healthcare organisations. Before it was sent out, the questionnaire was subjected to a pilot test to verify the clarity of the questions. Three Chief Executive Officers of three large public hospitals were involved in the pilot test. The research focused on those working in different positions (physicians, nurses, veterinarians, psychologists, etc.) both with and without a managerial role. Those individuals who have a managerial role also have a clinical background. The distinction between organisation role with managerial function (manager) and organisational role without managerial function (professional) reveals whether and how position influences the perception of key competences, given that the perspective of the respondent is parallel with the position they fill. In the healthcare sector, previous research has already highlighted the need to analyse data by distinguishing between managers and non-managers [14, 36].

In addition to position held, the questionnaire investigates specific managerial competences for each topic. Each respondent was asked to identify the most relevant item; in other words, the specific competence considered most important for healthcare professionals filling a managerial role was identified. The results of the survey were elaborated and presented in two parts, each with a defined goal.

In the first part, the focus is on the items most and least frequently chosen. The aim is to identify those specific competences perceived as being most relevant and those perceived as being least relevant. There is also a comparison between responses from managers and responses from professionals. In the second part, the focus shifts to analysing the sections for each topic. The aim is to verify for each topic how the competences should be used in practice. There is also a comparison between managers and professionals.

Data from the questionnaire were processed using IBM SPSS Statistics V25.0.

In order to identify statistically significant differences between groups (managers vs professionals), the Pearson’s chi-squared test was run using a 5% significance level (*p*-value ≤0.05). To evaluate the intensity of the relationship we used the Phi index, when both variables were dichotomous (manager/professional vs sections), and Cramer’s V, when only one of the two variables was dichotomous (manager/professional vs items). We used the categorisation proposed by Dancey and Reidy [37] to classify the strength of the association: “weak” with a Phi index (or Cramer’s V) between 0.1 and 0.3, “moderate” between 0.4 and 0.6 and “strong” between 0.7 and 0.9.

### Ethical approval

The present study was not submitted to an institutional ethics committee since this is not required under Italian legislation. All survey respondents gave their written consent to participate after being informed about the study.

## Results

### Managerial competences: topics and sections

From SLR, a total of 102 papers were initially identified and when we eliminated duplicates, 89 articles remained. We then read abstracts to eliminate articles that were not considered relevant for our research, and fifty-six articles were eliminated. The thirty-two remaining articles were read in their entirety. In this step, articles were read, analysed, and summarised, which also provided an additional quality control stage. The final number of studies included in the SLR was twenty-two. In the articles selected, we found fifteen different domains of managerial competences. However, we decided to include in our study only those topics that were reported at least by half of the articles read, and thus identified eight topics. Table 1 shows the fifteen topics, the articles that include each topic as relevant to manager-clinicians, and the percentage of articles that report that topic of the total of twenty-two articles analysed.

**Table 1:** Managerial competences: topics

Topic	Articles	%
Leadership	[25–27, 30, 38–53]	90.9%
Costing	[25–27, 30, 38–44, 46, 47, 49–52, 54, 55]	86.4%
Analysis	[25, 26, 30, 38, 40, 42–48, 50–55]	81.8%
Communication	[26, 27, 30, 38, 39, 41–50, 53–55]	81.8%
Human resource management	[25–27, 30, 39, 40, 43, 44, 47–51, 53–55]	72.7%
Organisational design	[25–27, 30, 38–40, 42, 44, 46–49, 51, 53, 55]	72.7%
Programming	[25–27, 30, 38, 40, 42–44, 47–49, 52, 54]	63.6%
Quality	[26, 27, 30, 39, 40, 43, 44, 46, 47, 50, 52, 54, 55]	59.1%
Change management	[25, 26, 44, 47, 51, 52, 55]	31.8%
Strategic planning	[26, 39, 43, 44, 47, 52, 54]	31.8%
Legality and ethics	[30, 39, 42, 43, 52, 55]	27.3%
Marketing	[26, 27, 30, 40, 43]	22.7%
Networking	[27, 40, 44, 47, 52]	22.7%
Problem solving	[30, 42, 43, 47, 54]	22.7%
Teaching	[40, 45, 46, 53]	18.2%

The eight topics were then discussed in the focus group in order identify the specific competences required of the manager-clinician. At least four specific competences for each topic emerged from the FGD, for a total of thirty-six competences which constitute the items of the questionnaire. Finally, in each topic, items were categorised into two sections according to how each competence is used in practice, as reported in Table 2.

**Table 2:** Managerial competences: sections

Topic	Section	Description
Leadership	*Hierarchic*	*•* Top-down leadership approach
	*Empowerment*	*•* Participatory leadership
Costing	*Broad*	*•* Economic evaluation at the macro-level
	*Sectorial*	*•* Economic evaluation at the micro-level
Analysis	*Operation management*	*•* Focus on practices to create efficiency
	*Project management*	*•* Focus on methods to achieve the project objectives
Communication	*Informal*	*•* Not using formal methods
	*Formal*	*•* Official exchange of information
Human resource management	*Basic approach*	*•* Focus on the basic aspects of HRM
	*Competence approach*	*•* Focus on enhancing the skills of employees
Organisational design	*Mechanistic structure*	*•* Bureaucratic vision of the organisation
	*Organic structure*	*•* Objective and results orientation
Programming	*Tools*	*•* Orientation to programming tools
	*Processes*	*•* Process planning orientation
Quality	*Managerial*	*•* Focus on management aspects that generate quality
	*Technical*	*•* Assessment of the technical aspects of quality

### Item analysis

The observation period yielded 585 questionnaires. Of the respondents, 298 were managers (50.94%) and 287 were professionals (49.06%).

Data analysis reveals that both groups (managers and professionals) shared similar opinions. The items considered most important, attracting more than 50% of preferences, were the same for both groups. The values for the categories were as follows: Defining goals according to available resources (manager 56.7% vs professional 51.9%); Planning organisation and processes (54.3% vs 49.5%); Making assessments by taking account of efficiency, efficacy, and quality (73.8% vs 69.0%); Interpersonal communication (66.4% vs 58.2%); and Creating a collaborative atmosphere (57.4% vs 62.7%).

Similarly, for the competences considered least relevant, attracting ≤5% of preferences, assessments made by the manager and professional groups were similar: Contributing to setting up reward benefit system (manager 4.0% vs professional 6.3%); Project design (5.7% vs 4.5%); Drafting procedures (1.7% vs 6.6%); Planning logistics in specific sectors in local areas (0.7% vs 1.4%); Planning user flows (4.7% vs 2.4%); Using information flows (3.4% vs 4.2%); Interpreting annual balance sheet (4.0% vs 8.4%); and Leading projects (4.7% vs 7.32%).

The Pearson’s chi-squared test found a different distribution of responses between the two groups only for the topic of “Quality” (*p*-value: 0.000). The value of Cramer’s V reveals an association, although of weak intensity (Cramer’s V: 0.196). Both groups consider “the assessment of clinical outcomes” to be the most relevant in this topic, although significantly more so for managers than for professionals: 40.0% vs 30.6%. However, in second position, managers placed “the identification of quality indicators for their organisational unit” (25.4%) while it was only in third position for professionals (11.1%). Professionals placed second “the evaluation of behaviour based on quality standards” (18.1%).

### Sections analysis

Analysing the occurrence of competences in everyday practice (see “Section” reported in Table 2, second column), in almost all areas of inquiry, one section predominated over the other. With the exception of two topics (“Analysis” and “Organisational Design”), all topics showed one section with at least twenty percentage points fewer than the other (see Table 3).

**Table 3:** Section distribution for each topic

Topic	Section	Manager	Profes-sional	Overall	Phi index
Leadership	*• Hierarchic*	33.9%	32.1%	33.0%	0.020
	*• Empowerment*	66.1%	67.9%	67.0%	
Costing	*• Broad*	11.4%	15.7%	13.5%	0.062
	*• Sectorial*	88.6%	84.3%	86.5%	
Analysis	*• Operation management*	47.0%	38.7%	42.9%	0.084
	*• Project management*	53.0%	61.3%	57.1%	
Communication	*• Informal*	77.2%	71.8%	74.5%	0.062
	*• Formal*	22.8%	28.2%	25.5%	
Human resource management	*• Basic approach*	24.4%	30.0%	27.2%	0.061
	*• Competence approach*	75.5%	70.0%	72.8%	
Organisational design	*• Mechanistic structure*	37.6%	43.6%	40.5%	0.061
	*• Organic structure*	62.4%	56.4%	59.5%	
Programming	*• Tools*	34.9%	35.9%	35.4%	0.010
	*• Processes*	65.1%	64.1%	64.6%	
Quality	*• Managerial*	28.2%	43.6%	35.7%	0.160^*^
	*• Technical*	71.8%	56.4%	64.3%	

^*^p-value ≤5%

The three topics where one section is much more frequently indicated than the other included: “Costing”, where competences were seen to involve the capacity to make cost assessment of projects (86.5%); “Communication”, where the capacity to communicate informally was considered to be of key importance (74.5%); and “Human Resource Management”, where management of colleagues based on competences was considered to be very relevant (72.8%). These data are essentially confirmed by examining separately the opinions of management and professionals (Table 3). The two groups actually expressed similar judgements; the biggest difference was for “Quality”, where there were more than fifteen percentage points difference. The Pearson’s chi-squared test confirmed that there is a different orientation between managers and professionals in this topic (*p*-value: 0.000). The Phi index indicates the existence of a weak association (Phi index: 0.160). Managers, in fact, focused significantly more on technical quality than did professionals (71.8% vs 56.4%). The greatest similarities occurred for “Leadership” (1.8 percentage points of difference) and “Programming” (1.0 percentage points). Table 3 shows the evaluations of managers, professionals and the whole sample for each section.

## Discussion

This paper has investigated managerial competences required by professionals for the role of manager-clinician. The research reveals the point of view of both professionals and managers. Our results are consistent with the work of Demou, Lalloo and Macdonald [36], which did not reveal major differences between managers and professionals. The only area that shows differing perceptions by the two groups is the topic of “Quality”.

Managers show a more technical approach towards quality, which for them refers to individual services or specific results (quality of outcomes, standardized professional behaviour, specific risks). On the other hand, professionals show a managerial approach to quality, which for them refers to the organizational conditions necessary to generate quality service (quality indicators for their organisational unit, quality plan for the unit, and attention to the motivation of professionals) [56]. This might seem like a reversal of roles.

However, it should be noted that professionals have a more balanced view of the two sections of quality (43.6% Managerial vs. 56.4% Technical; See Table 3) compared to managers. It appears that professionals, alongside their interest in techniques, also feel the need for organizational rules that make it easier to pursue quality objectives. On the other hand, the strong technical orientation of managers may reflect the fact that they believe they can make a full contribution and successfully play their role where specific objectives are defined and results carefully evaluated.

However, in general, our findings suggest that the perception of the competences necessary for those in a managerial role is more closely linked to processes (a set of interrelated activities to achieve a result), than to position (filling a managerial or other type of role). An explanation could be that those working in a healthcare organisation all perceive problems in a similar way, regardless of the role they fill, and collectively consider certain competences necessary for improving performance.

Our results also have important implications for HRM practices in public healthcare organisations. Rondeau and Wagar highlight that certain HRM practices can lead to better organisational performance in the public health sector [57] and many other authors have stated that HRM should be geared towards enhancing human capital [13]. The enhancement of human capital, especially in organisations with a strong presence of professionals, cannot be separated from the recognition and development of competence, experience and knowledge [2, 58]. In this sense, our work provides a clear picture of which competencies require particular attention in the recruitment / selection, training, evaluation and awarding phases, although it is limited to managerial competences. These results can support policy makers and public managers who want to set up positive HRM practices for healthcare professionals. In fact, public organisations and government policies should work together to retain and develop the competences considered as priorities for the healthcare sector [13]. While governments should promote system-level policies which lie above bureaucratic personnel management, public managers need to be able to successfully implement these innovative forms of management [2, 9].

Finally, the theoretical contribution of our work is twofold. First, since many public organisations are coming under pressure to revise their human resource policies in the face of changing labour markets, some implications for retention professionals in the public health sector can be drawn. The failure of NPM suggests there is a need for innovative HRM practices, especially for professional-based organisations. An HRM approach able to recognise and enhance employees’ competences could be an important strategic lever for public organisations. Our exploratory study could be preliminary to subsequent research that goes in this direction. Secondly, although the issue of managerial skills in healthcare is widely discussed in literature, findings are often fragmentary and inconsistent [25, 26]. Our work includes a SLR that can be used as a starting point for more empirical studies.

## Conclusion

The public sector has often been criticised for its bureaucratic personnel management system, and over the years, the emphasis on the adoption of private sector HRM practices has increased. However, in recent years, research has begun to recognise the limitations of importing private HRM practices into the public sector, especially into organisations with a strong presence of professionals, as in the case of healthcare organisations [3, 9]. Historically, clinicians, as compared with other professional groups, are more inclined to reject managerial intervention in their work, and the direct control of their behaviour by the organisation has often been ineffectual [17]. This means that public healthcare organisations need to develop HRM practices that are able to enhance the autonomy and competences of these professionals [59]. In doing this, public organisations should be able to align the objectives and interests of employees to their own, thus enabling the achievement of better performance for the organisation [10, 60]. In most industrialised countries, the issue of competences in healthcare is complicated because clinical professionals in higher positions in the hierarchy are also required to possess managerial competences, on which however there is no shared vision. The aim of our study was to highlight the competences deemed most relevant for professionals who also hold a managerial role, thus providing a useful contribution to organisations that want to invest in their workers.

Nevertheless, our study possesses certain limitations. The first observation is that our findings may be influenced by the context of the Italian healthcare sector in which our research was carried out. Issues in healthcare organisation management will, of course, differ from country to country, so perceptions of competence relevance will vary accordingly. It should however be specified that some previous research has shown that in healthcare there is a universalistic approach to HRM practices and that those that are identified as best practices can also be valid in other contexts [13, 16]. The second limitation is that there may be further elements influencing responses to the questionnaire, which were not taken into account through our elaborations. Respondent characteristics such as age, gender, training, length of service, years of service as manager, and the number of organisations worked at are all factors that may have influenced the responses given in our sample. However, in this case too, similar studies have shown that these characteristics do not statistically significantly modify the respondent’s perception of what key competences in healthcare are [36].

## Supplementary information

**Additional file 1.** Questionnaire on managerial competences, Questionnaire administered online to healthcare workers in order to identified the specific competence considered as most important for healthcare professionals filling a managerial role.

## Data Availability

The datasets analysed during the current study are available from the corresponding author on reasonable request.

## Abbreviations

HRM: Human resource management
NPM: New Public Management
NHS: National Health System
SLR: Systematic Literature Review
FGD: Focus group discussion
