# Supplementary material for: Managerial competences in public organisations: the healthcare professionals’ perspective
Source: BMC Health Serv Res. 2020 Apr 15;20:303. doi: 10.1186/s12913-020-05179-5 (PMC7158078; doi:10.1186/s12913-020-05179-5)
Supplement: Supplementary file 1 — Additional file 1. Questionnaire on managerial competences, Questionnaire administered online to healthcare workers in order to identified the specific competence considered as most important for healthcare professionals filling a managerial role. [file 12913_2020_5179_MOESM1_ESM.docx]

**Additional file 1**

**QUESTIONNAIRE ON MANAGERIAL COMPETENCES**

**Organisation role**

□ Healthcare professional with managerial function

□ Healthcare professional without managerial function

**Profession**

Please, specify your profession (i.e. physicians, nurses, veterinarians, psychologists, etc.): __________________

**For each group of statements, identify the specific competence considered as most important for healthcare professionals filling a managerial role.**

**1. Managerial competences (1/8)**

□ Defining employees’ characteristics

□ Contributing to setting up reward benefit system

□ Setting up competence development education for colleagues

□ Defining professional competences’ need of a specific unit

**2. Managerial competences (2/8)**

□ Designing organisation roles and assigning specific responsibilities

□ Project design

□ Drafting procedures

□ Defining goals according to available resources

**3. Managerial competences (3/8)**

□ Defining tools to support health professionals in their activities

□ Defining coordination tools to overcome issues

□ Planning logistics in specific sectors in local area

□ Planning user flows

□ Planning daily activities

□ Planning organisation and processes

**4. Managerial competences (4/8)**

□ Interpreting complex phenomena using various indicators. (Data and trend analysis)

□ Assessing project sustainability

□ Building performance indexes to assess responsibilities

□ Using information flows

**5. Managerial competences (5/8)**

□ Interpreting annual balance sheet

□ Assessing the organisation and economic impact of technologies

□ Making assessments by taking account of efficiency, efficacy, and quality

□ Assessing activities costs and profits

**6. Managerial competences (6/8)**

□ The identification of quality indicators for their organisational unit)

□ The definition of a quality plan for the unit)

□ The risk assessment (clinical risk, corruption risk, etc.)

□ Assessing colleague satisfaction

□ The assessment of clinical outcomes

□ The evaluation of behaviour based on quality standards

**7. Managerial competences (7/8)**

□ Communicate with the press

□ Public speaking

□ Written communication (reports, presentations, social medias, etc.)

□ Interpersonal communication

**8. Managerial competences (8/8)**

□ Leading projects

□ Creating a collaborative atmosphere

□ Leading teams

□ Negotiation skills
